# Neural Representation Learning for Compact and Efficient Modeling of Monte Carlo Phase Space Data

**DOI:** 10.1016/j.ijpt.2026.101949

**Published:** 2026-06-28

**Authors:** Serdar Charyyev, Cynthia Chuang, Yong Yang, Lei Xing, Lianli Liu

**Affiliations:** Department of Radiation Oncology, Stanford University, Palo Alto, CA 94305, USA

**Keywords:** Monte Carlo, Phase space data, Neural representation learning, Implicit neural networks, Protons

## Abstract

**Purpose:**

Monte Carlo (MC) simulations provide gold standard dose calculations in radiation therapy but generate large phase space (PHSP) files that limit clinical implementation. We developed NeRP-MC, the first neural representation learning approach for PHSP data modeling, and evaluated its ability to model particle distributions from minimal training data.

**Materials and Methods:**

We investigated proton PHSP modeling at 242 and 140 MeV. For both energies, a reference proton pencil beam PHSP containing 25 million particles was generated using TOPAS. A multi-layer perceptron with Fourier feature encoding was trained to predict particle energies from spatial and momentum inputs. We evaluated NeRP-MC in 3 scenarios: 1) compact energy modeling given full spatial and momentum information, 2) energy modeling from sparse PHSP data (1.25 million particles, 20-fold reduction), and 3) replacing the PHSP with parametric Gaussian spatial/momentum distributions and network-predicted energies conditioned on the Gaussian-sampled inputs. Validation used in-water dose distributions compared via gamma index analysis.

**Results:**

The trained network requires only 600 KB for storage versus 3 GB for the original PHSP and predicts 25 million particle energies in under 0.5 seconds on an NVIDIA A100 GPU. NeRP-MC generated energies showed close agreement with reference data across all 3 scenarios. Depth-dose profiles, lateral profiles, and penumbra regions were accurately reproduced. Gamma pass rates exceeded 99% at 3%/2 mm and 90% at the strictest 1%/1 mm criterion.

**Conclusion:**

NeRP-MC offers compact modeling and fast prediction of particle energies from particle spatial and momentum information and promises to replace the large-scale PHSP with a parametric Gaussian model of spatial and angular variables and the NeRP model of particle energy variables. NeRP-MC has the potential to advance MC simulation efficiency for radiation therapy through a substantial reduction in computational and storage requirements while maintaining dosimetric accuracy.

## Introduction

Monte Carlo (MC) methods have become increasingly indispensable in medical physics, particularly in radiation therapy and medical imaging. Their usage has grown exponentially over recent decades,^1^ driven by the need for accurate dose calculations in increasingly complex treatment scenarios. This transition towards MC methods as the preferred algorithm for dose calculation in treatment planning is evidenced by numerous Task Group (TG) reports published by the American Association of Physicists in Medicine, including TG157, TG195, TG186, and TG105, among others.^2^, ^3^, ^4^, ^5^

The prominence of MC methods is particularly justified in radiation therapy, where they are considered the gold standard for determining dose distributions within patients.^6^ This is especially crucial in proton therapy, where the finite range of protons and their complex interactions with matter necessitate highly accurate dose calculations.^7^, ^8^, ^9^, ^10^ Proton therapy offers distinct advantages over conventional photon radiotherapy, including superior dose conformity and reduced integral dose to healthy tissues, making it especially valuable for treating deep-seated tumors and pediatric cases.^11^ The growing adoption of proton therapy worldwide^12^ has further emphasized the need for accurate and efficient dose calculation methods.

However, the clinical implementation of MC methods faces significant challenges, primarily due to their computational demands. The necessity to simulate individual particle transport through complex geometries and track millions of histories to achieve acceptable (<2%) statistical uncertainty requires substantial computational resources, even with modern high-performance computing capabilities. To address these limitations, vendors and researchers often provide phase space (PHSP) data, which stores essential information such as 3D position, direction, and energy at specified planes.^13^ While PHSP data enables the calculation of various dosimetric quantities without simulating the entire beamline,^14^, ^15^ it presents its own challenges. For instance, a single PHSP file for a 6 MV beam with a 10 × 10 cm² field at the jaws exit plane can occupy approximately 30 GB of storage. When considering multiple energies, field sizes, and planes, the storage requirements become prohibitive for practical sharing and utilization. Because of these challenges, researchers have explored various methods to obtain PHSP data more efficiently. For example, Wang et al^16^ developed an algorithm for automatic generation of PHSP for proton therapy, achieving high accuracy in dose calculations with gamma-index pass rates above 96% using 0.6%/0.6 mm criteria.

Recent advances in deep learning,^17^ particularly in neural networks capable of learning complex high-dimensional data distributions, offer even more promising solutions to these challenges. A significant breakthrough came with the application of generative adversarial networks (GANs)^18^ to compact beam source modeling in MC simulations. Sarrut et al^19^ pioneered this approach, developing a method to compress large PHSP files into compact neural networks of approximately 10 MB, achieving less than 1% voxel-wise relative difference in energy deposition compared to reference distributions. This work was extended to SPECT imaging applications,^20^, ^21^ demonstrating the versatility of GAN-based approaches. Subsequently, several researchers have successfully applied GANs to various aspects of medical physics simulation, including optical MC simulations,^22^ kV X-ray imaging,^23^ PET-Linac modeling,^24^ and MV X-ray Linac PHSP generation.^25^ However, GAN is known to be difficult to train, and the model was only evaluated to predict PHSP data that is of the same size as the training PHSP data. The scalability of the model to larger PHSP dataset remains unclear.

A particularly promising development that has emerged to address these challenges is implicit neural representation learning (NeRP).^26^, ^27^ Successful application of NeRP to various radiotherapy data, including medical images,^28^, ^29^ treatment plans^30^ and linear accelerator beam profiles^31^, ^32^ was demonstrated. Building upon these advances, we propose to develop a NeRP model for compact modeling of PHSP data from sparse training samples. Our approach aims to address the fundamental challenges of PHSP data storage and generation while maintaining the accuracy necessary for clinical applications. The successful development of such a model could lead to a neural-network-based fast dose prediction^33^ tool suitable for clinical use as either a primary or secondary dose calculation engine.

Beyond compact PHSP data modeling, this research enables transformative clinical applications, including real-time adaptive treatment planning and rapid dose verification for quality assurance. The methodology extends naturally to other radiation therapy modalities, including photon and electron therapies. By learning accurate dose distributions from limited training data, this approach could facilitate personalized treatment planning and support the clinical translation of novel treatment techniques.^34^, ^35^, ^36^, ^37^, ^38^, ^39^, ^40^

## Materials and methods

### Overview of the workflow

**Figure 1:**
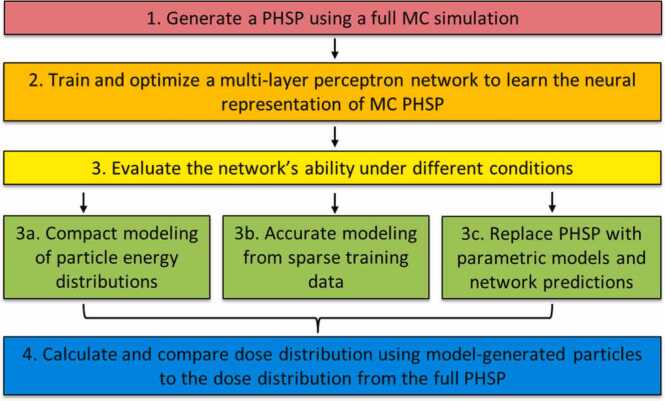
Workflow of the proposed methodology.

Figure 1 outlines the workflow of our methodology. Step 1 involves performing a full MC simulation of Varian ProBeam nozzle using Tool for Particle Simulation (TOPAS)^41^ to obtain reference PHSP data containing particle position (x, y, z), type, energy, and momentum (dx, dy), and weight at a specific plane. For this proof-of-concept study, we focused exclusively on primary protons. Secondary particles (neutrons, photons, etc.) were not scored at the PHSP plane. All protons in the PHSP had unity weight (w = 1.0). We chose 2 energies: 140 and 242 MeV single spot proton pencil beam PHSPs containing details of 25 million particles, recorded at the isocenter plane in ASCII and binary formats. The obtained PHSP serves as the ground truth for subsequent steps.

Step 2: A multi-layer perceptron (MLP) network was trained to learn the neural implicit representation of the MC PHSP. Specifically, the network was designed to model the conditional inverse cumulative distribution function (CDF) of particle energy given particle spatial and momentum parameters. After training, the network predicts particle energy for a given set of spatial and momentum inputs by sampling from the learned inverse CDF. Because the inverse CDF is implicitly defined through the functional relationship between network inputs and outputs, learning a neural network that parameterizes such an implicitly defined function is referred to as NeRP. Unlike GAN-based approaches that learn the distribution of data values directly, NeRP models a continuous mapping from dataset coordinates to data values, where each coordinate–value pair constitutes a training sample. This coordinate-based formulation enables efficient learning of intrinsic data structure without requiring large-scale datasets.

We investigated network training with full-size PHSP dataset, as well as sparse sampling (20 folds/1.25 million particles). The trained network was evaluated for compact modeling of particle energy distributions with spatial and angular information (Step 3a), modeling of full-scale particle energy distributions with sparsely sampled training data (Step 3b), and replacing PHSP with a Gaussian parametric model of particle spatial and angular distribution and network-modeled particle energy distribution (Step 3c). For this proof-of-concept, we chose the independent Gaussian sampling estimated from the original PHSP. To quantify the fidelity of the surrogate PHSP model learnt by the neural network, dose distributions calculated using model-predicted particle information were compared to the one using full PHSP (Step 4) using the gamma index.

### Network architecture and model training

Similar to previous work on NeRP,^28^, ^31^, ^32^ an MLP network M was implemented to learn the function defined over the PHSP dataset Φ:v→E. The input v consists of the normalized particle radial position $r=\sqrt{x^{2}+y^{2}}\in \left(0,1\right)$, normalized momentum magnitude $m=\sqrt{u_{x}^{2}+u_{y}^{2}}\in \;\left(0,1\right)$ and a quantile variable u that represent the empirical conditional CDF value of particle energy given $\left(r,m\right)$. During network training, the input r and m were each partitioned into 10 equal count bins. Within each bin, particle energies were sorted in ascending order, and the quantile variable was assigned as $u=(\mathrm{Rank}+1)/\left(n+1\right)$, where n denotes the number of particles in that bin. The quantile variable was further transformed using a logit function before being input to the network.

The network consists of 4 fully connected layers, each with 256 neural nodes followed by a periodic activation function. To effectively capture high-frequency components of the dataset, Fourier feature mapping F was used to encode the input dataset coordinates:

(1) $$F\left(v\right)=\;\left[\cos\left(2\pi \mathrm{Bv}\right),\sin\left(2\pi \mathrm{Bv}\right)\right]$$

before the coordinates were input to the network, where entries of the matrix **B** were sampled from Gaussian distribution $N\left(0,\sigma^{2}\right).W$e experimentally set the size of the Fourier feature encoding (number of rows for B) as 256 and the scale σ as 4. The MLP network parameters θwere optimized using an Adam optimizer with a learning rate of 1e-4 for 500 iterations. L2 loss between network-predicted particle energy and the ground truth PHSP particle energy was minimized across all the particles included in the training dataset:

(2) $$\theta^{*}=\arg\min\sum_{i=1}^{N}{\left\|M_{\Phi }\left(F\left(v_{i}\right)\right)-E_{i}\right\|}_{2}^{2}$$

### Model evaluation

Step3: After training, given a set of particle spatial and momentum parameters, particle energy can be predicted by drawing u from a uniform distribution between 0 and 1 and performing a single forward pass through the trained network. Each particle in the PHSP was characterized by 5 parameters: energy (E), position coordinates (x, y), and direction cosines (dx, dy). The z-coordinate was excluded since the PHSP was recorded in a plane (isocenter). We evaluated the trained network under 3 experimental scenarios:

Test (A) – Compact particle energy modeling. Instead of saving energy information of individual particles, the optimized neural network was used to predict the energy of an arbitrary particle, given its spatial and momentum information from the original full PHSP. We compared network-predicted particle energies to the reference PHSP data, as well as the size of network parameters θ* to the size of individual particle energy data.

Test (B) – Particle energy modeling with sparse training data. We further investigated NeRP of PHSP with sparse training data. Full-scale particle energy information was predicted from the trained network with full PHSP particle spatial and angular parameters, including those not in the training dataset and compared to the full PHSP.

Test (C) – Replace PHSP with parametric modeling: In this test, we simulated spatial and angular variables from parametric Gaussian distribution models and used the trained network to predict particle energies from the simulated inputs. We compared the particle descriptors generated from the Gaussian model and the NeRP network to the ground truth PHSP data.

Step 4: Model-predicted particle information was evaluated through 2 approaches. First, we compared marginal distributions of all particle parameters between model-generated and full PHSP. Second, we assessed dose distributions calculated using model-generated particles against those from the full PHSP, utilizing absolute dose difference, percent dose difference, and gamma index analysis to quantify the fidelity of the surrogate PHSP representation learned by the neural network.

### Comparison of dose distributions

Dose distributions were calculated using TOPAS in a water phantom of 10 × 10 × 40 cm dimensions, which is discretized into 1× 1 × 1 mm voxels and situated such that there is an air gap of 5 cm between the surface of the water phantom and the PHSP starting plane. The beam is a single pencil beam spot incident at the center of the x-y surface of the water phantom. Gamma analysis was performed using 3%/2 mm, 2%/1 mm, and 1%/1 mm criteria. The 3%/2 mm criterion, while originally derived from photon IMRT QA (TG-218),^42^ serves as a clinically established baseline for dosimetric comparisons; the tighter 2%/1 mm and 1%/1 mm criteria were additionally applied to reflect the stricter tolerances appropriate for MC dose calculation benchmarking in a homogeneous geometry. Reference distribution is the dose calculated with full 25 million particle MC PHSP, and evaluated distribution is the dose calculated with NeRP-MC generated PHSP. Dose threshold of 10% of the maximum dose was used. Global normalization was used, with the normalization point set to the maximum dose of the reference distribution. 3D volumetric gamma analysis was implemented using Matlab (The MathWorks, Inc, Natick, MA). A gamma index value γ ≤ 1 indicates passing (agreement within tolerance), while γ > 1 indicates failing. We report both pass rates (percentage of voxels with γ ≤ 1) and gamma value distributions.

## Results

The optimized network requires only 600 KB for parameter storage, as compared to the 3 GB (if ASCII format) or 875 MB (if binary format) full-size PHSP data. Using an NVIDIA A100 GPU, the model demonstrated efficient performance by predicting energy information for 25 million particles in under 0.5 s

**Figure 2:**
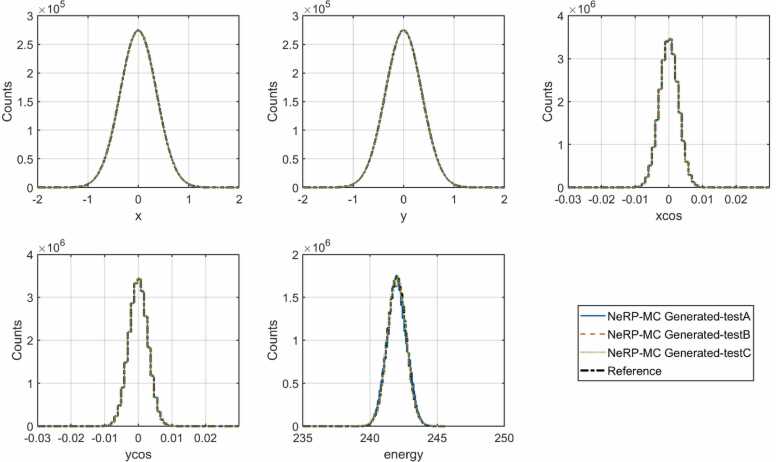
Comparison of 242 MeV proton PHSP distributions between the reference data (black dashed line) and NeRP-MC-generated data for 3 test scenarios: (test A, blue line) compact particle energy modeling, (test B, red line) particle energy modeling with sparse training data, and (test C, green line) particle prediction with Gaussian descriptors of particle spatial and angular information.

Figure 2 presents a comprehensive comparison of the 5 PHSP parameters—x, y, dx, dy, E—for the 3 test scenarios (tests A, B, and C). In all subplots, the distributions generated by NeRP-MC for all 3 scenarios (solid-colored lines) show nearly perfect overlap with the reference PHSP data (black dashed line). Notably, the consistency across tests A, B, and C demonstrates that the model’s performance remains robust regardless of whether it is predicting from full-sized data, sparse data, or parameter-based distributions.

**Figure 3:**
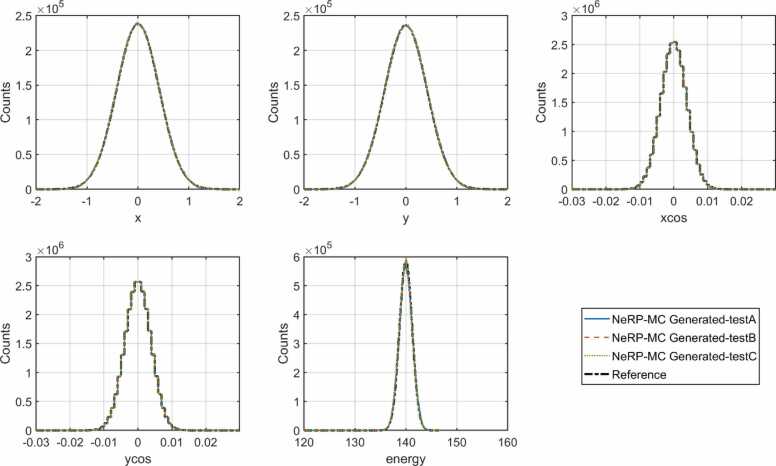
Same layout as Figure 2, but for 140 MeV.

Similarly, Figure 3 displays the PHSP analysis for a 140 MeV proton beam, demonstrating the model's adaptability to different energy levels. As observed in the 242 MeV case, the NeRP-MC generated distributions exhibit high fidelity to the reference data across all 3 test scenarios.

**Figure 4:**
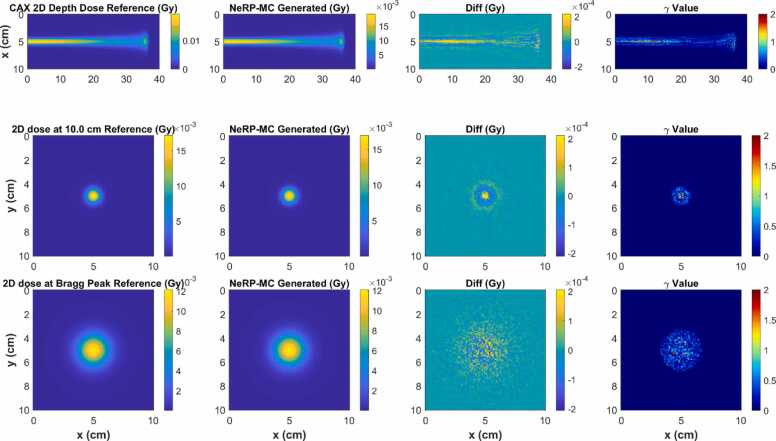
Comparison of dose distributions for test A of 242 MeV protons. Top row: CAX view. Middle row: transverse slice at 10 cm depth. Bottom row: Bragg peak region. From left to right: reference dose, NeRP-MC generated dose, absolute dose difference, and gamma index analysis 1%/1 mm.

Figure 4 compares the dose distributions calculated using the model-generated (test A) and original PHSP files for the 242 MeV beam. The figure shows 3 key depths: the central axis (CAX) view (top row), a transverse slice at 10 cm depth (middle row), and the Bragg peak region (bottom row). For each depth, we present the reference dose (1st column), NeRP-MC generated dose (2nd column), absolute dose difference (3rd column), and gamma index analysis (last column) with stringent 1%/1 mm criteria. The difference maps show minimal deviations in most regions.

**Figure 5:**
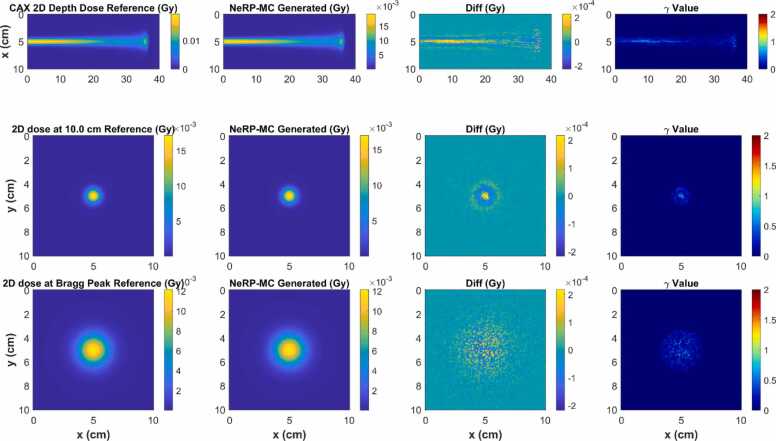
Same layout as Figure 4, but for test B with gamma criterion of 2%/1 mm.

**Figure 6:**
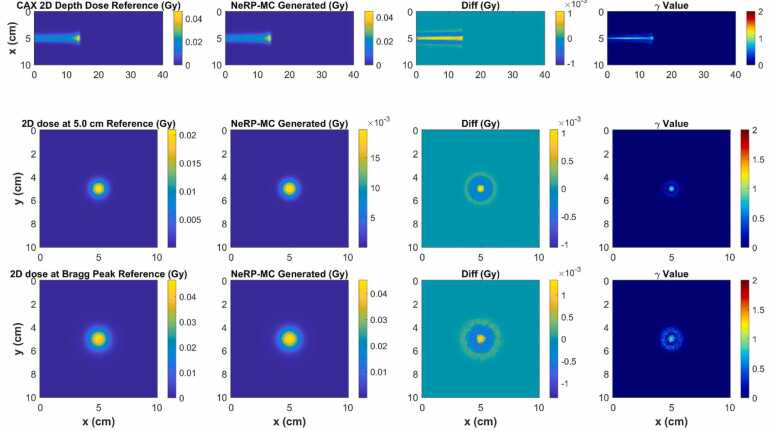
Same layout as Figure 4, Figure 5, but results are shown for test C of 140 MeV with the gamma criterion of 3%/2 mm. Middle row shows the transverse slice at 5 cm depth.

Figure 5 illustrates the performance for the 242 MeV proton beam under test B (particle energy modeling with sparse training data) using a 2%/1mm gamma criteria, while Figure 6 replicates this analysis for the 140 MeV beam under test C (particle prediction with Gaussian descriptors of particle spatial and angular information) using a 3%/2mm criteria. In both figures, the 3 primary planes show high agreement between reference and NeRP-MC generated dose distributions, with difference maps remaining small across all regions.

**Figure 7:**
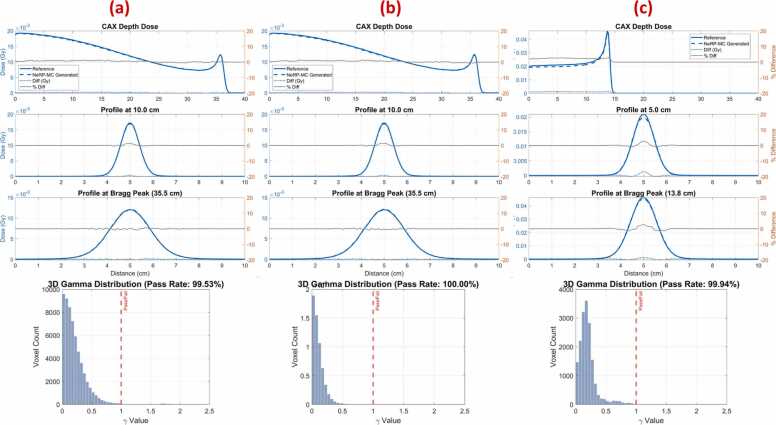
1-D dose profiles and 3D gamma index distributions for 3 evaluation scenarios: (a) 242 MeV test A (1%/1 mm), (b) 242 MeV test B (2%/1 mm), and (c) 140 MeV test C (3%/2 mm), corresponding to the configurations detailed in Figure 4, Figure 5, Figure 6, respectively. Rows 1–3: CAX depth dose, lateral profile at mid-depth (10 cm for 242 MeV; 5 cm for 140 MeV), and lateral profile at Bragg peak. Solid blue lines represent the reference dose, dashed blue lines represent the NeRP-MC predicted dose, and dotted blue lines show the absolute dose difference (left y-axis, Gy). The grey lines indicate the percentage difference (right y-axis). Row 4: 3D Gamma distribution histograms for each respective volume, with the red dashed line indicating the pass/fail threshold (γ = 1).

Figure 7 presents 1-dimensional dose profiles and corresponding volumetric gamma distributions. Columns (a), (b), and (c) correspond to the configurations detailed in Figure 4 (242 MeV, test A), 5 (242 MeV, test B), and 6 (140 MeV, test C), respectively. The top 3 rows compare the reference dose (solid blue line), NeRP-MC generated dose (dashed blue line), and absolute dose difference (dotted blue line) against the primary y-axis (left, in Gy), while the percent difference (solid grey line) is shown on the secondary y-axis (right). The agreement is good across all profiles. The bottom row displays the 3D gamma distribution histograms for each scenario, showing pass rates of 99.53%, 100.00%, and 99.94% for tests A, B, and C, respectively. These high pass rates confirm that the model consistently generates acceptable dose distributions.

Table 1 summarizes the dosimetric comparison between the reference and the NeRP-MC-generated PHSPs across all 3 test configurations for 242 and 140 MeV beams. For 242 MeV, Bragg peak depth, distal fall-off, and spot FWHM values agree within 0.2 mm across all tests, and gamma pass rates are at or near 100% for all 3 criteria, including the strictest 1%/1 mm. For 140 MeV, spatial metrics agree within 0.7 mm, and gamma pass rates remain around 99%, 97% and 90% for the 3%/2 mm, 2%/1 mm, 1%/1 mm criteria, respectively.

**Table 1:** Dosimetric comparison between reference and NeRP-MC-generated PHSP for 242 and 140 MeV proton beams.

Energy	Metric	Reference (cm)	NeRP-MC (cm)		
			Test A	Test B	Test C
242 MeV	Bragg Peak Depth	35.78	35.78	35.78	35.78
	Distal fall-off	0.56	0.56	0.57	0.54
	Spot FWHM at 10 cm	0.95	0.96	0.96	0.96
	Spot FWHM at Bragg Peak	2.08	2.07	2.06	2.07
	Gamma Pass Rate (%)	3%/2 mm	100	100	100
		2%/1 mm	100	100	100
		1%/1 mm	99.5	99.6	99.6
140 MeV	Bragg Peak Depth	13.67	13.67	13.67	13.67
	Distal fall-off	0.37	0.37	0.37	0.37
	Spot FWHM at 5 cm	1.08	1.15	1.15	1.15
	Spot FWHM at Bragg Peak	1.35	1.39	1.39	1.40
	Gamma Pass Rate (%)	3%/2 mm	99.9	99.9	99.9
		2%/1 mm	96.9	97.2	97.3
		1%/1 mm	90.5	89.9	89.9

Results are shown for 3 independent NeRP-MC test configurations (Test A, B, and C). FWHM stands for full width at half maximum.

## Discussion

In this study, we developed NeRP-MC, a compact NeRP model for proton PHSP data. The compact parameter footprint represents a practical reduction over binary PHSP files, and the sub-second inference time enables workflows that were previously impractical with file-based approaches.^16^ Compared to previous studies,^19^ the network does not require a full-size PHSP for training: accurate energy predictions were achieved from as few as 1.25 million particles (20-fold sparse sampling), making NeRP-MC well-suited for scenarios where acquiring a dense reference PHSP is impractical.

Particle energy distributions were well-reproduced with minor deviations appearing only in the low-energy tail of the spectrum, involving a few hundred particles out of 25 million (<0.01%), confined to low-dose regions and producing no measurable impact on dose distributions in the clinically relevant high-dose volume.

The dosimetric results (Table 1) provide rigorous validation of NeRP-MC across both energies and all 3 test configurations. The slight reduction in gamma pass rates at the tighter 1%/1 mm criterion for 140 MeV relative to 242 MeV likely reflects the sharper dose gradients in 140 MeV proton beams as well as the broader relative energy spread of 140 MeV, which may not be captured with full granularity by the current network architecture. Critically, dosimetric accuracy was maintained across all 3 test scenarios, including Test C, where the model had no access to original particle coordinates, confirming that the NeRP approach is robust to the level of input approximation.

Test C, in which spatial and angular coordinates were sampled from independent Gaussian distributions and energies were predicted by the network, merits particular attention. This is the most clinically relevant scenario, as it replaces the PHSP file entirely, eliminating the need to store or transfer particle coordinates, with only the Gaussian distribution parameters and the compact network weights. Despite the additional approximation introduced by Gaussian sampling, dose distributions for both energies remained well within acceptable agreement with reference calculations, as shown in Figure 6, Figure 7 and Table 1. We note that the current implementation uses independent (uncorrelated) Gaussian models for each PHSP variable, which is a simplification. Correlations between spatial and angular variables exist in real beams, and future work will investigate whether incorporating multivariate Gaussian or NeRP-based models for the spatial/angular variables further improves accuracy, particularly for more complex beam configurations.

Several limitations of this study should be acknowledged. While we tested two proton energies (140 and 242 MeV) and demonstrated consistent performance across both, the evaluation was limited to a single nozzle geometry (Varian ProBeam), a simple pencil beam configuration, and a homogeneous water phantom. Validation in heterogeneous geometries and across a broader clinical energy range will be necessary before clinical deployment. The model also currently handles only primary protons with unity weight; extension to secondary particles and weighted sampling will be required for general-purpose PHSP modeling. Additionally, the Gaussian approximation of spatial and angular distributions may be insufficient for beams with strongly non-Gaussian profiles, such as those modified by range shifters or patient-specific hardware.

Taken together, these results demonstrate that NeRP-MC provides a compact, fast, and accurate approach to PHSP energy modeling. By combining NeRP-MC predicted energies with parametric descriptions of spatial and angular variables, it offers a practical path toward eliminating large PHSP files in proton therapy workflows, with direct implications for adaptive planning, quality assurance, and broader access to MC-based dose calculation.

## Conclusions

In this work, we developed a compact model for MC PHSP using NeRP techniques. The model accurately predicts particle energy distributions given spatial and angular information and promises to replace PHSP data files when combined with Gaussian parametric models of spatial and angular variables. The model requires only a negligible fraction of the space for parameter storage and demonstrates scalability when trained with sparse data samples. The success of this approach opens several promising avenues for future work. Primary among these is the further refinement of the model architecture and training methodology to enhance its accuracy and generalization capabilities. Additionally, we plan to extend this framework to photon PHSP modeling, which could have important implications for radiation therapy treatment planning and quality assurance. These developments could potentially lead to more efficient and accessible MC simulations in medical physics applications.

## CRediT authorship contribution statement

**Serdar Charyyev:** Conceptualization, Methodology, Software, Formal Analysis, Data Acquisition, Validation, Visualization, Writing - original draft. **Cynthia Chuang:** Conceptualization, Resources, Writing - review and editing. **Yong Yang:** Conceptualization, Resources, Supervision, Writing - review and editing. **Lei Xing:** Conceptualization, Methodology, Software, Writing - review and editing, Supervision, Funding acquisition, Project administration, Resources. **Lianli Liu:** Conceptualization, Methodology, Software, Formal analysis, Data acquisition, Validation, Visualization, Project administration, Writing - original draft.

## Declaration of Conflicts of Interest

The authors declare that they have no known competing financial interests or personal relationships that could have appeared to influence the work reported in this paper.
